# Sessional Lectures

**Published:** 1899-05-06

**Authors:** 


### SESSIONAL LECTURES.

Clinical lectures at the National Hospital for the Paralysed and Epileptic, Queen Square, W.C., free to gentlemen who are practitioners or students, will be held at half-past three on the following Tuesdays: May 9th, Dr. Beevor, "Myopathies"; May 16th, Dr. Ferrier, F.R.S., "Disseminated Sclerosis"; May 23rd, Dr. Ormerod, "Hysteria"; May 30th, Sir W. Gowers, F.R.S., "Epilepsy"; June 6th, Dr. Tooth, "Neuritis" (lantern demonstration); June 13th, Mr. Horsley, F.R.S., "Surgery of the Nervous System"; June 20th, Mr. Ballance, "Surgery of the Nervous System"; June 27th, Dr. Risien Russell, "Combined Degenerations of the Spinal Cord" (lantern demonstration); July 4th, Dr. James Taylor, "Electrical Testing"; July 11th, Mr. Marcus Gunn, "Ocular Paralyses."

